# Matching phosphorylation response patterns of antigen-receptor-stimulated T cells via flow cytometry

**DOI:** 10.1186/1471-2105-13-S2-S10

**Published:** 2012-03-13

**Authors:** Ariful Azad, Saumyadipta Pyne, Alex Pothen

**Affiliations:** 1 Department of Computer Science, Purdue University, West Lafayette, IN 47906, USA; 2 Department of Medical Oncology, Dana-Farber Cancer Institute, Harvard Medical School, Boston, MA 02115, USA; 3 Broad Institute of MIT and Harvard University, Cambridge, MA 02142, USA

## Abstract

**Background:**

When flow cytometric data on mixtures of cell populations are collected from samples under different experimental conditions, computational methods are needed (a) to classify the samples into similar groups, and (b) to characterize the changes within the corresponding populations due to the different conditions. Manual inspection has been used in the past to study such changes, but high-dimensional experiments necessitate developing new computational approaches to this problem. A robust solution to this problem is to construct distinct templates to summarize all samples from a class, and then to compare these templates to study the changes across classes or conditions.

**Results:**

We designed a hierarchical algorithm, **flowMatch**, to first match the corresponding clusters across samples for producing robust meta-clusters, and to then construct a high-dimensional template as a collection of meta-clusters for each class of samples. We applied the algorithm on flow cytometry data obtained from human blood cells before and after stimulation with anti-CD3 monoclonal antibody, which is reported to change phosphorylation responses of memory and naive T cells. The **flowMatch **algorithm is able to construct representative templates from the samples before and after stimulation, and to match corresponding meta-clusters across templates. The templates of the pre-stimulation and post-stimulation data corresponding to memory and naive T cell populations clearly show, at the level of the meta-clusters, the overall phosphorylation shift due to the stimulation.

**Conclusions:**

We concisely represent each class of samples by a template consisting of a collection of meta-clusters (representative abstract populations). Using **flowMatch**, the meta-clusters across samples can be matched to assess overall differences among the samples of various phenotypes or time-points.

## Background

In multiparametric flow cytometry, fluorophore-conjugated antibodies are used to measure the expression of multiple markers expressed on the surface and within the interior of cells in a given sample. As cells flow in a fluid stream, excitation by laser causes the fluorochrome with the attached antibodies to emit light of a specific wavelength, and the detected fluorescence intensity gives a measure of the identity and amount of expression of the different markers per cell [1]. Thus a flow cytometric **sample **consists of the quantitative measurement of particular cell characteristics such as its lineage, state or function in terms of the expression of the selected markers under the given experimental conditions. It can be represented as a matrix of size *N *× *d*, where *N *(typically in the range 10^3^-10^5^) is the number of cells, and *d*, the number of markers measured in the experiment, also called the dimension of the sample, is in the range 4-20. For a given set of markers, a **population **is a group of cells in a sample with similar marker expression, and thus biologically similar to other cells within the group but distinct from those outside the group. Identifying cell populations in a sample (traditionally a manual process known as "gating") is a well-studied clustering problem that summarizes a sample with population parameters [2]. From an algorithmic point of view, populations may be described as multivariate **clusters **of *d*-dimensional points where each cluster is represented by its distribution parameters such as location (mean, mode), variance, size, etc. In this paper we will use the term population and cluster interchangeably depending on the context.

**Figure 1:**
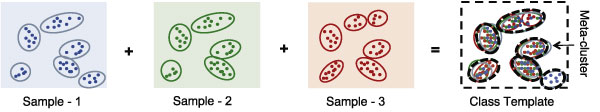
**Example**. Meta-clusters and a class template: Construction of a class template by merging three samples. Cells are denoted with dots, clusters with solid ellipses and meta-clusters with dashed black ellipses.

The idea of characterizing a sample - a collection of cells - with clusters can be extended to characterizing a cohort or **class **- a collection of samples - with a higher-level technical description. Towards this end, we assume that samples belonging to a particular class show homogeneous clustering, by which we mean that at least some of the populations correspond from sample to sample. Such clusters have small distances from corresponding clusters in other samples of that class, when an appropriate measure of distance is used. We note that clusters from samples belonging to different classes might not have such correspondences. Under this assumption, we can summarize the samples of a class with a **class template **by algorithmically merging similar populations from different samples into **meta-clusters**. A **meta-cluster **is an abstract population formed by grouping together similar cell populations across samples, and the **template **of a class is the collection of all the meta-clusters constructed in this manner. For convenience, an illustration of these concepts is shown in Figure 1.

Recently developed computational methods for flow cytometry have focused on automating gating via clustering [3], but not on the equally important problem of constructing and matching meta-clusters for heterogeneous samples. Here we report the design of a hierarchical algorithm, **flowMatch**, to construct class templates consisting of a set of meta-clusters summarizing a set of samples from a particular class, and to study how these meta-clusters change under different experimental conditions.

The ability to systematically characterize a class of multi-dimensional samples with a well-defined template - in terms of its meta-cluster components - is useful in applications of flow cytometry. In addition to providing a cogent description of the core population structure that is shared among samples within a class, the templates also allow an objective way to assess the overall differences in those structures across classes. Here we applied the concepts of template and meta-cluster in quantifying tyrosine phosphorylation differences in different CD4^+ ^T cell populations in a cohort of thirty human subjects, before and after stimulation of whole blood with anti-CD3 monoclonal antibody (mAb) [4]. Previous studies have shown that different T cell subsets (naive, memory, effector) show different overall phosphorylation responses upon stimulation [4-7]. In these approaches each sample was gated to identify cell populations of interest, and pairs of samples, before and after stimulation, were compared to detect the phosphorylation responses. However, Maier et. al. reported that the autoimmune disease-associated allele at CTLA4 gene on chromosome 2q33 alters phosphorylation responses in naive and memory T cells [4]. Thus depending on the genetic profile of the subject, samples may differ in their response upon stimulation. It is therefore challenging to summarize the phosphorylation responses of all the samples in a class from manual observations of the phosphorylation effect in individual samples. In this setting, the samples belonging to each class (*pre-stimulation *and *post-stimulation *classes in this paper) can be described by a class template, and by matching meta-clusters across templates we can better assess the population-specific effects of the stimulation experiment.

To address the problem of systematic template construction, we designed and developed an agglomerative hierarchical meta-clustering algorithm, **flowMatch**, that is similar in spirit to the well-known UPGMA clustering algorithm [8,9]. The algorithm begins with clustered samples obtained from a Dirichlet Process Mixture (DPM) Model as described in [10]. It then proceeds hierarchically by selecting the closest pair of samples, merging them into an intermediate template, and repeating until all the samples of a class are merged into a final template consisting of a set of meta-clusters for the class. After computing the class templates, we match meta-clusters from one template to another using a generalized edge cover in an underlying bipartite graph [10].

In a recent approach, FLAME, Pyne et. al. [11] formed class templates from many samples of a particular class by first clustering the locations of all clusters using Partitioning Around Medoids (PAM). Then they refined the clusters by comparing each sample with the templates using an integer programming formulation of a weighted *b*-matching in a bipartite graph. The templates of the two classes (pre-and post-stimulation) were then compared by using weighted bipartite matching. However, this approach has a few drawbacks. First, in a collection of heterogenous samples, multiple templates might be necessary to effectively model the whole class, whereas FLAME tries to form a single PAM-based template for a class assuming limited heterogeneity. A hierarchical merging of samples can tackle the problem by using a threshold value to create a merge forest (instead of a merge tree). Second, FLAME uses within-class Euclidean distances between the mode of a cluster and the medoid of a meta-cluster as edge weight, ignoring the underlying distributions. To address this, we used Kullback-Leibler divergence from the distributions as a dissimilarity measure, which takes into account both the location and the shape of the distributions. Third, in their bipartite *b*-matching formulation, FLAME uses the population size as the capacity of a vertex to match multiple clusters to a meta-cluster. This could cause a problem if the number of cells within clusters varies widely across the samples. Finally, possible outlier populations present in only a few samples seem to be ignored by their model. In contrast, we use a generalized edge cover (GEC) formulation [10], where a meta-cluster is allowed to match with zero, one, or multiple clusters from the other class, while minimizing a suitable objective function.

## Results and discussion

### Dataset

We analyzed raw flow cytometric data generated *ex vivo *by Maier et. al. originally intended to determine the allelic variation of the phosphorylation responses [4]. In their work, whole blood samples from thirty subjects was stained using phospho-site-specific labelled antibodies against CD4, CD45RA, SLP76 (pY128), and ZAP70 (pY292), before and after stimulation with anti-CD3 antibody. Of these antibodies, CD4 and CD45RA were used to differentiate among different T cell subpopulations, and the tyrosine phosphorylation sites SLP76 and ZAP70 were used to detect the effect of stimulation. Following the convention of Maier et al., we used the CD4^+ ^clusters CD4^+^CD45RA^high ^and CD4^+^CD45RA^low ^to represent the naive and memory T cell populations respectively [4,12]. (Here a '+' symbol means that a marker is expressed, 'high' means that it is expressed at a high level; similarly, 'low' means that a marker is expressed at a low level, and a '*-*' symbol means that it is not expressed at all. Thus both these T cell populations have the CD4 marker expressed, and the naive T cell population has a higher expression level corresponding to CD45RA relative to the memory T cell population.)

### Clustering

We consider each sample as a mixture of Gaussian components and used Dirichlet Process Mixture (DPM) Model for the clustering [13,14]. One reason for selecting DPM over other clustering methods is its ability to detect the number of clusters automatically. We used a publicly available Matlab implementation of DPM clustering by Teh [15]. We observed that the DPM clustering was not able to obtain well-separated clusters in several samples. We are currently exploring the use of other clustering methods for the entire data set. Meanwhile, here we choose to study in detail a subset of ten samples for which well-separated clustering structure could be obtained. We also report the results from all thirty samples and compare them with the ten well-clustered samples in our discussion.

### Class templates

**Figure 2:**
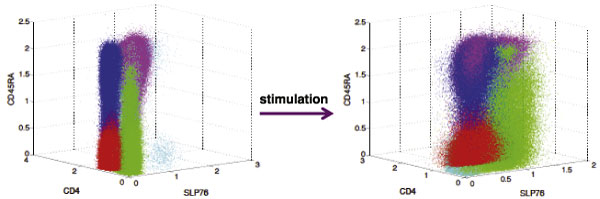
**Templates**. Structures of templates: Templates from ten well-clustered samples before (left) and after (right) stimulation (projection into three dimensions, ZAP70 is not shown). In a template, each meta-cluster is indicated with a distinct color; four meta-clusters are present in each template. Corresponding meta-clusters across templates are plotted with the same color. Naive CD4^+^CD45RA^high ^T cell population is colored in blue, and Memory CD4^+^CD45RA^low ^T cell population is colored in red. Details of the clusters in each meta-cluster are shown in Table 1.

We first construct templates from the ten well-clustered samples before stimulation (pre-stimulation template) and after stimulation (post-stimulation template). The two templates are shown in Figure 2. The average number of clusters present in a sample before stimulation is 6.3, and after stimulation it is 7.1. The **flowMatch **algorithm identifies six meta-clusters in both templates, indicating that the algorithm does not over-split or merge populations within a class. However, the small difference in the number of meta-clusters is because **flowMatch **combines a few clusters within a sample that were possibly incorrectly split by the initial DPM clustering.

Table 1 describes the membership of clusters from the original samples among the different meta-clusters. Each row in the Table corresponds to a meta-cluster. Each sample usually has a representative cluster in most meta-clusters, which confirms the presence of similar populations across samples (e.g., sample 7 has clusters present in the five meta-clusters before stimulation and in all six meta-clusters after stimulation). However, **flowMatch **is flexible in placing none, one, or more than one cluster from a sample into meta-clusters, reflecting the absence of a particular population or the presence of one or more populations (due to splitting in initial clustering phase) in a sample. Some of the meta-clusters in the Table include two clusters from some samples, and one includes three clusters from one sample; the meta-cluster in the last rows has clusters from few samples. This flexibility gives the algorithm the opportunity to correct the initial clustering via the meta-clustering process. Notice also that the stimulation with anti-CD3 monoclonal antibody changes the composition of the meta-clusters in subtle ways.

**Table 1:** Composition of meta-clusters

Type	Meta-clusters before stimulation	Meta-clusters after stimulation
Memory T cell	1-10	1-2, 3^+^, 4-10

Naive T cell	1-3, 4^+^, 5-7, 8^+^, 9-10	1-3, 4^+^, 5-10

	1^+^, 2, 3^+^, 4^+^, 5-10	1, 2^+^, 3, 4^+^, 5^+^, 6^+^, 7^+^, 8-10

	1-4, 5^+^, 6^+^, 7-10	1^+^, 2-4, 5^+^, 6^+^, 7^+^, 8^+^, 9^++^, 10

	1-6, 7^+^, 8^+^, 9^+^, 10	

	1-4, 8	1, 2^+^, 3-6, 7^+^, 8-10

		1, 5-9

The composition of each meta-cluster: Each entry denotes the list of samples that contribute a cluster to a particular meta-cluster. A '+' in the superscript of a sample *s *indicates that multiple clusters from *s *appear in a meta-cluster (e.g., *s*^++ ^means three clusters from sample *s *appear in that meta-cluster.) Matching meta-clusters across templates are shown in same row, and the known cell population types are also shown.

### Effect of stimulation on phosphorylation responses

**Figure 3:**
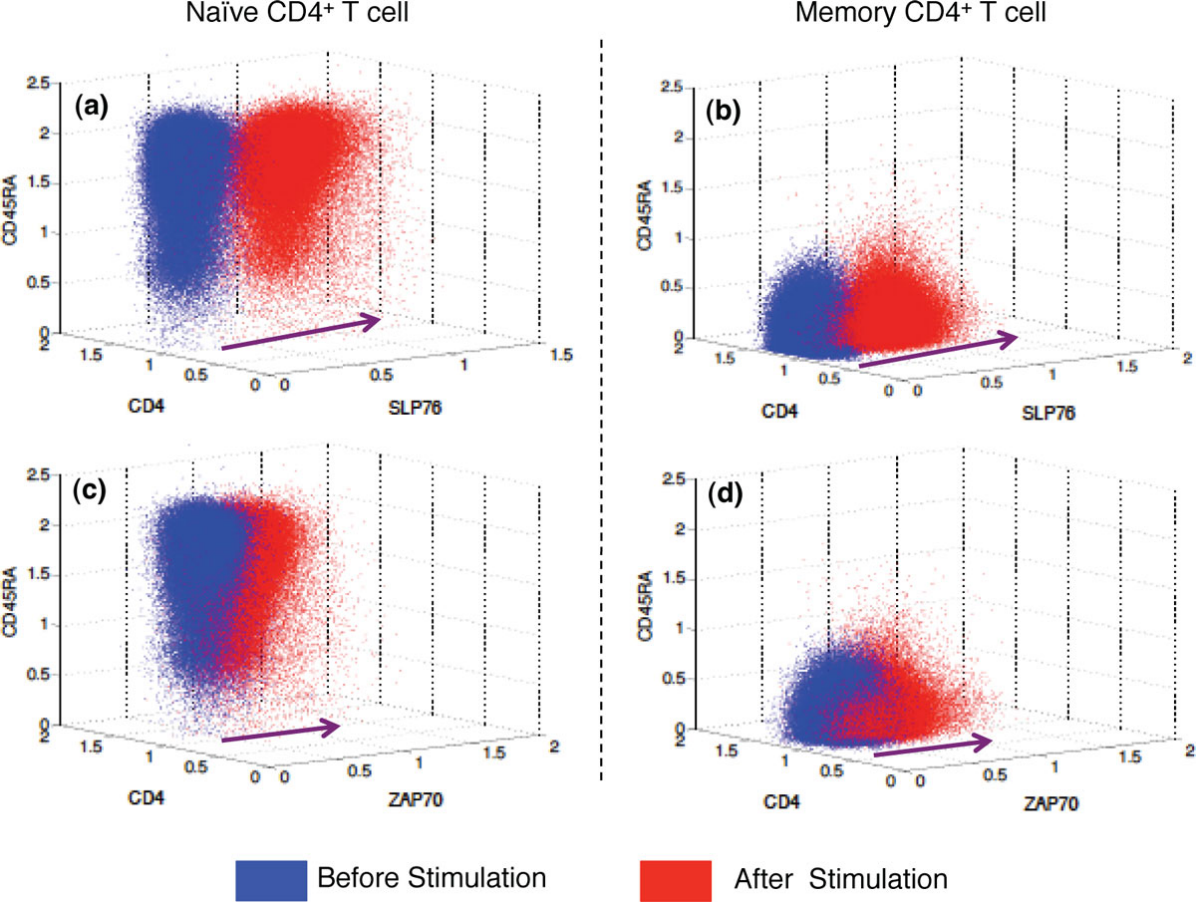
**Stimulation**. Templates before and after stimulation: Detection of increased phosphorylation (shown with arrows) in naive CD4^+^CD45RA^high ^(left column) and Memory CD4^+^CD45RA^low ^(right column) T cell populations at the meta-cluster level before (blue) and after (red) stimulation. Top row (subfigures (a), (b)) shows the effect on SLP76 and the bottom row (subfigure (c), (d)) shows the effect on ZAP70.

From Figure 2, most CD4^+ ^T cell populations display overall increased SLP76 phosphorylation upon stimulation with anti-CD3 antibody, which agrees with the observations in [4,11]. Similar results were obtained for ZAP70. We consider meta-clusters representing naive CD4^+^CD45RA^high ^and Memory CD4^+^CD45RA^low ^T cell populations, and show the effect of stimulation on these subsets in Figure 3. Previous studies performed with individual samples [4,11] reveal that memory CD4^+ ^T cells display a lower baseline SLP76 phosphorylation level than naive CD4^+ ^T cells. Our approach obtains this result with the entire class of samples (Figures 3(a) and 3(b)), by identifying corresponding meta-clusters between the two class-templates. We also notice the low phosphorylation shift in ZAP70 residues (Figures 3(c) and 3(d)) relative to the SLP76 residues upon stimulation. This demonstrates that **flowMatch **can match corresponding meta-clusters across templates despite the variable amount of phosphorylation shifts.

### Stability of templates

**Figure 4:**
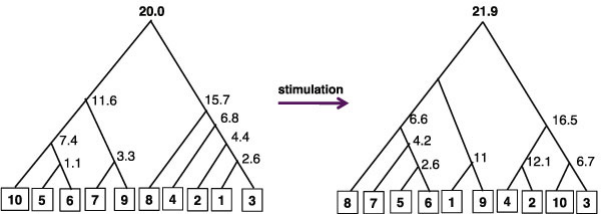
**prePostTree**. The pre-stimulation and post-stimulation merge trees: The hierarchy of sample-merges to construct a template from ten well-clustered samples before (left) and after (right) stimulation. The leaves denote original samples and are marked with numerical labels. The internal nodes indicate sub-templates with the final template at the root. The cost of forming a sub-template (the merge cost of two children) is also shown at each non-leaf node.

The **flowMatch **algorithm builds a binary merge forest with the class templates at the roots of the trees, by merging a pair of samples or sub-templates at a time. Let *T *be a sub-template (or template) constructed by merging *T*_left _(left child) and *T*_right _(right child). Then, *C_T_*, the cost of the template *T*, is the cost of merging *T*_left _and *T*_right_, which is the cost of the generalized edge cover, given in Equation 1 (see the Methods section). We show the merge tree for the templates before and after stimulation in Figure 4, and indicate the cost associated with each internal node in that tree.

**Figure 5:**
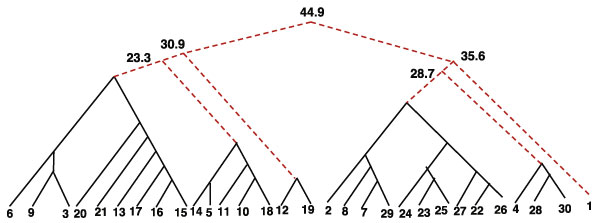
**fullTree**. The merge forest of thirty samples: The hierarchy of sample-merges to construct templates from all thirty samples after stimulation. Numerical labels are used to denote samples at the leaves. Unstable sub-templates are indicated with broken red lines. The costs of the unstable sub-templates are also shown in their corresponding nodes.

A large template cost indicates heterogeneity among the samples participating in the template construction. How much heterogeneity (merge cost) should we tolerate while building a template? We argue that any pair of samples taken from different classes (pre- and post-stimulation) should be more heterogenous than a pair of samples in the same class. For example, when we consider the ten samples that are well-clustered, the pre-stim template and post-stim template costs are respectively 20 and 21.9 in Figure 3, whereas the minimum cost of merging any pair of cross class samples is 31.3. We call the former **stable templates **since they compactly represent all samples in the class. However, if we construct a single template from all the thirty samples (Figure 5 for post-stimulation) the cost is approximately 45 for both pre-stimulation and post-stimulation templates, whereas the minimum cost of merging any pair of cross-class samples is 23*:*3. This suggests that we need more than one template to represent all of the samples. We use the minimum merge-cost among all cross class pairs as a cut-off value for all thirty samples to decompose the merge tree into a merge forest, and declare all sub-templates constructed up to that point as the templates of the cohort of samples. In Figure 5 we denote unstable merges by broken red edges, and removing those edges will produce six templates for the thirty samples.

## Conclusions

We concisely represent each class of samples by a template consisting of a collection of meta-clusters (representative abstract populations). We have developed a hierarchical template formation algorithm, **flowMatch**, that is able to construct representative class templates in the presence of tolerable variations in the samples. Using **flowMatch**, the meta-clusters across samples can be matched to assess overall differences among the samples of various phenotypes or time-points.

## Methods

### Problem description

Consider *N *samples *S*_1_, *S*_2_,...*S_N _*belonging to a class *A*, where each sample is individually clustered. Let the *i*th sample consist of *n_i _*clusters, $S_{i}=\left\{c_{1},c_{2},.\;.\;.\;,c_{n_{i}}\right\}$. From the *N *samples we wish to construct a class template *T_A _*with *K *meta-clusters {*M*_1_, *M*_2_,..., *M_K_*}, where *K *≥ 1. The *i^th ^*meta-cluster *M_i _*consists of *k_i _*clusters from the samples. If *k_i _*= 1, a meta-cluster is an original cluster from one of the samples. In this setting, every cluster from all samples is uniquely assigned to some meta-cluster. After constructing a template *T_A _*for class *A *and a template *T_B _*for class *B*, we wish to match meta-clusters from the two class templates.

### Formation of templates: the flowMatch algorithm

We use a bottom-up pairwise merging of samples to construct class templates. The procedure constructs a binary forest, where initial samples are leaves, and the roots of the trees are the class templates for the subset of samples in each tree. If there is only one tree in the forest, then all the samples have one class template. In Figure 4 we show the trees for the pre-and post-stimulation samples. The internal nodes of the tree consist of meta-clusters from subsets of samples and we refer to them as **sub-templates**. A high level description of the template construction procedure is given below:

#### 1. Initialize

Consider each sample as a sub-template with each cluster as meta-cluster. Construct the set of current sub-templates  for each sample. Calculate the similarity between every pair of samples (as explained below).

#### 2. Merge closest pair

Find the most similar pair of sub-templates *T *and *T*' from  and merge them into a new sub-template $\tilde{T}$. Remove *T *and *T*' and add $\tilde{T}$ to . If  or the cost of merging is too high, stop and return  as the final template(s).

#### 3. Calculate similarity

Calculate the similarity of  to all other sub-templates in , and return to Step 2.

##### Merging a pair of sub-templates

The building block of the procedure is the merging of a pair of sub-templates. Consider merging two sub-templates *T *= {*m*_1_, *m*_2_,..., *m_K_*} and $T^{\prime }=\left\{m_{1}^{\prime },m_{2}^{\prime },\;.\;.\;.\;,m_{K^{\prime }}^{\prime }\right\}$, where *T *has *K *meta-clusters and *T' *has *K*' meta-clusters. We calculate the dissimilarity between a pair of original clusters *c *and *c*' with the average Kullback-Leibler (KL) divergence (also known as relative entropy) from their corresponding distributions [16]. We then define the dissimilarity between two meta-clusters *m *= {*c*_1_, *c*_2_,..., *c_k_*} and $m^{\prime }=\left\{c_{1}^{\prime },c_{2}^{\prime },.\;.\;.,c_{k^{\prime }}^{\prime }\right\}$ as the average of the distance between each pair of clusters:

$$d\left(m,m^{\prime }\right)=\left(1/)|m||m^{\prime }|\right)\underset{c_{i}\in m}{\sum }\underset{{c^{\prime }}_{i}\in m^{\prime }}{\sum }d\left(c_{i},{c^{\prime }}_{j}\right).$$

We model the sub-template pair *T *and *T*' by a complete bipartite graph *G *= (*T*, *T*', *E*), where every meta-cluster *m_i _*∈ *T *is a vertex in one part, every meta-cluster $m_{j}^{\prime }\in T^{\prime }$ is a vertex in the other part, and each pair of vertices $\left\{m_{i},m_{j}^{\prime }\right\}$ is joined by an edge with weight $w\left(\left\{m_{i},m_{j}^{\prime }\right\}\right)=d\left(m_{i},m_{j}^{\prime }\right)$. We compute a minimum weight generalized edge cover [10] in the bipartite graph *G *= (*T*, *T*', *E*). A generalized edge cover (GEC) is a subset of uncovered vertices *V_uc _*⊂ *T *∪ *T*' and a subset of edges such that each vertex *m *∉ *V_uc _*has at least one edge incident on it while minimizing the objective function:

(1) $$min\left(\underset{\left\{m_{i},{m^{\prime }}_{j}\right\}\in GEC}{\sum }d\left(m_{i},{m^{\prime }}_{j}\right)+\lambda *|V_{uc}|\right).$$

**Figure 6:**
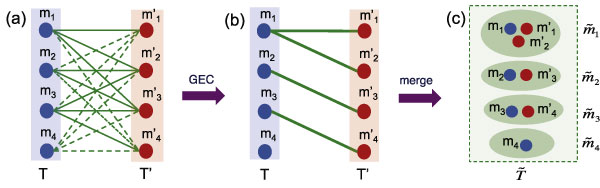
**GEC**. Generalized edge cover: The use of generalized edge cover to merge two sub-templates *T *and *T*'. (a) The bipartite graph model from two sub-templates. A pair with high dissimilarity is connected with a broken edge. (b) The generalized edge cover (GEC) solution which matches each vertex to zero or more vertices on the other side. The solution tries to avoid the broken edges. (c) The sub-template obtained from the GEC solution by merging matched vertices.

Here *λ *is a free parameter that represents the penalty for leaving a vertex uncovered, and is used to control the number of such vertices. The generalized edge cover matches each vertex to zero or more vertices in the other part while avoiding edges with high weights. In the generalized edge cover, we merge all the meta-clusters (vertices) matched together into a single meta-cluster. Figure 6 shows an example that merges two sub-templates into a new one. Notice that a vertex not covered in the generalized edge cover (the vertex *m*_4 _in *T *in Figure 6) is included in a a meta-cluster by itself, indicating the presence of a cluster in only one sub-template. All newly formed meta-clusters constitute the merged sub-template $\tilde{T}$, which then replaces the two templates *T *and *T*' in .

##### Order of merging

We pick a pair of sub-templates to merge such that the cost of merging them is the minimum among all possible pairs in current list . The cost of merging a pair of sub-templates *T *and *T*' is the cost of the generalized edge cover computed using with the bipartite graph model shown in Equation 1.

## Comparison of templates

After constructing two class templates we again use a generalized edge cover to match meta-clusters across classes.

### The time complexity of the flowMatch algorithm

Let *N *be the number of samples and *K *be the maximum number of meta-clusters among all sub-templates generated by the **flowMatch **algorithm. The similarity between a pair of sub-templates can be computed in time *O*(*K*^3 ^log *K*) [10]. The initialization step requires *O*(*N*^2^) similarity computations. The hierarchical merging step needs *O*(*N*) iterations, with each iteration computing *O*(*N*) similarity measures. Hence the time complexity of the algorithm is *O*(*N*^2^*K*^3 ^log *K*).

The algorithm runs under a minute on a 2.4 GHz PC for our dataset with *N *= 30 and *K *= 11.

## Authors' contributions

Pyne and Pothen conceived the study; Azad and Pothen designed the meta-template creation algorithm; Pyne guided the statistical aspects of the work; and Azad implemented the algorithms. All three authors participated in writing the paper.

### Competing interests

The authors declare that they have no competing interests.
